# When should we discontinue antiarrhythmic therapy for atrial fibrillation after abdominal surgery?

**DOI:** 10.1186/1471-2482-13-S1-A35

**Published:** 2013-09-16

**Authors:** Gennaro Pagano, Dario Leosco, Nicola Ferrara, Nicola Rocco, Corrado Rispoli, Loredana Iannone, Serena Testa, Rita Compagna, Antonello Accurso, Bruno Amato

**Affiliations:** 1 Department of Translational Medical Sciences, Federico II University of Naples, Naples, Italy; 2 “Salvatore Maugeri” Foundation, IRCCS Scientific Institute of Telese Terme, Benevento, Italy; 3 Department of Biomedical, Surgical and Dental Sciences, University of Milan, Milan, Italy; 4 Department of General Surgery - ASL NA1, Cardinale Ascalesi Hospital, Naples, Italy; 5 Department of General Surgery, Fatebenefratelli Hospital, Benevento, Italy; 6 Department of General, Geriatric, Oncologic Surgery and Advanced Technologies, Federico II University of Naples, Naples, Italy

## Aim of the study

The purpose of this study was to determine whether the duration of antiarrhythmic therapy after discharge from the hospital following abdominal surgery is related to the incidence of atrial fibrillation (AF) recurrence in elderly patients with the occurrence of peri-operative AF.

## Background

The occurrence of peri-operative AF after abdominal surgery is a clinical condition burdened by several complications, especially in the elderly [1-3]. When AF is successfully converted to sinus rhythm, it is unlikely to recur, and nearly all of these patients are discharged from the hospital in sinus rhythm. It is not clear how soon these patients may discontinue antiarrhythmic therapy to avoid drugs side effects without risking recurrence of AF.

The recurrence of AF needs different kind of treatment. Medical therapy includes various antiarrhythmic drugs to control heart rate and restore sinus rhythm and anticoagulation to reduce the tromboembolic risk [4]. Overactivation of sympathetic nervous system, related to surgery stress [5], could be reduced by the treatment with antiarrhythmic drugs, such as Beta Blockers, and may reduce the incidence of AF recurrence [6-10].

## Methods

A pilot study was conducted in 19 elderly patients (age > 65 years) who underwent abdominal surgery (right emicolectomy, sigmoidectomy and anterior rectal resection) and with occurrence of peri-operative AF that successfully reverted to sinus rhythm. They were prospectively randomized at dismissal to receive antiarrhythmic therapy for 1 week (six patients in group A), 3 weeks (seven patients in group B), or 6 weeks (six patients in group C). Patients were followed up for an additional 4 weeks after discontinuation of antiarrhythmic therapy for detection of recurrence of AF.

## Results

There was no significant difference in the recurrence of AF among groups (0%, 2%, and 0% for groups A, B, and C, respectively).

## Conclusions

In elderly patients with peri-operative AF after abdominal surgery, converted to normal sinus rhythm before hospital discharge, have a benign course and the duration of antiarrhythmic therapy shorter than one week is appropriate.
